# A study of three-time-point dynamic changes in aPS/PT and standard aPL antibodies and their association with IVF pregnancy outcomes in APS-related recurrent pregnancy loss

**DOI:** 10.3389/fmed.2025.1738040

**Published:** 2026-01-12

**Authors:** Fenjian Lu, Penghao Li, Panyu Yang

**Affiliations:** 1 Department of Laboratory Medicine, and Department of Medical Education, Sichuan Jinxin Xinan Women’s and Children’s Hospital, Chengdu, China; 2 Center for Reproductive Medicine, The Third People’s Hospital of Chengdu, Chengdu, China

**Keywords:** anti-phosphatidylserine/prothrombin antibodies, antiphospholipid syndrome, IVF pregnancy outcome, recurrent pregnancy loss, standard antiphospholipid antibodies

## Abstract

**Objective:**

This study aimed to evaluate dynamic anti-phosphatidylserine/prothrombin (aPS/PT) and antiphospholipid (aPL) antibody changes in antiphospholipid syndrome-recurrent pregnancy loss (APS-RPL) patients and their relationship with pregnancy outcomes.

**Methods:**

This study included 100 RPL patients with APS features and 30 healthy women who had a normal delivery from 2020 to 2022. First, the differences in aPL and aPS/PT antibody levels between the study group and the control group were compared. Then, a mixed-design repeated measures analysis of variance (RM-ANOVA) was used to assess the time effects, group effects, and their interactions. Pairwise comparisons between time points within each group were performed using one-way repeated measures ANOVA. Finally, antibody levels at each time point were compared between the successful pregnancy group and the pregnancy loss group.

**Results:**

(1) The levels of aPS/PT immunoglobulin M (IgM) and anticardiolipin (aCL) IgG in the study group were significantly higher than those in the control group (*P* < 0.01). (2) In the successful pregnancy group, both aCL IgG and aPS/PT IgM levels were significantly lower after treatment and during early pregnancy compared to pre-treatment levels, and anti-β2-glycoprotein I (aβ2GPI) IgM was also significantly lower during early pregnancy compared to both pre-treatment and post-treatment levels (*P* < 0.01). (3) In the pregnancy loss group, aCL IgG was significantly lower after treatment and during early pregnancy compared to pre-treatment levels, but aPS/PT IgM and aPS/PT IgG were significantly elevated during early pregnancy (*P* < 0.01), showing completely opposite dynamic trends to the successful pregnancy group. (4) Between-group comparisons showed that the pregnancy loss group maintained higher levels of aPS/PT IgM after treatment and during early pregnancy, and aPS/PT IgG levels were also significantly higher during early pregnancy compared to the successful pregnancy group (*P* < 0.01).

**Conclusion:**

A post-treatment or early-pregnancy decline in aCL IgG, aPS/PT IgM, or aβ2GPI IgM is associated with successful pregnancy in APS-RPL patients, whereas early-pregnancy elevations in aPS/PT IgM and IgG are strongly linked to pregnancy loss. The distinct dynamic profiles of aPS/PT antibodies underscore their relevance to pregnancy outcomes, highlighting the value of longitudinal aPS/PT monitoring for identifying high-risk patients and informing individualized immunologic management.

## 1 Introduction

Recurrent pregnancy loss (RPL) is defined by the European Society of Human Reproduction and Embryology (ESHRE) as two or more consecutive pregnancy failures, with identifiable etiologies found in approximately 50% of affected individuals (1, 2). Epidemiological studies indicate that RPL affects up to 6% of women of reproductive age (3–5), with antiphospholipid syndrome (APS) recognized as one of the most important and identifiable causes. APS is an autoimmune disorder characterized by a hypercoagulable state, typically presenting with thrombotic events or obstetric complications, accompanied by the persistent presence of antiphospholipid (aPL) antibodies targeting phospholipid-binding proteins on cellular membranes (6–8). APS accounts for approximately 5–20% of RPL cases (2, 9).

According to the 2006 Sydney classification criteria, the diagnosis of APS requires at least one clinical criterion and one laboratory criterion, including persistent positivity for lupus anticoagulant (LA), anticardiolipin (aCL) IgG/IgM, or anti-β2-glycoprotein I (aβ2GPI) IgG/IgM (10). Clinical criteria encompass vascular thrombosis and a range of pregnancy morbidities, such as ≥3 consecutive miscarriages before 10 weeks of gestation in the absence of hormonal, anatomical, or chromosomal abnormalities; a single unexplained fetal loss after 10 weeks; or preterm delivery before 34 weeks due to severe preeclampsia, eclampsia, or placental insufficiency. However, recent studies have reported that some women who meet the clinical criteria for APS or experience RPL test negative for conventional aPL antibodies, a condition referred to as seronegative APS (11, 12), highlighting limitations of current laboratory markers.

Consequently, the search for new aPL antibodies with clinical relevance has intensified over the past decade. Among them, anti-phosphatidylserine/prothrombin (aPS/PT) antibodies have emerged as notable non-criteria aPL markers (11). Accumulating evidence indicates that aPS/PT antibodies serve as strong predictors of thrombotic events and obstetric complications (13–15), participate in the pathophysiology of APS (16, 17), and represent potential serological biomarkers for the disease (11). Nevertheless, their association with pregnancy outcomes remains controversial, with some studies failing to demonstrate significant correlations (18–20), while others have reported clear links between aPS/PT antibodies and miscarriage or pregnancy-related complications (19, 20).

Given these inconsistencies and the fact that the majority of previous studies relied on single time-point antibody measurements, the present study evaluated the dynamic changes in aPS/PT and standard aPL antibodies before treatment, after treatment, and during early pregnancy and examined their relationship with pregnancy outcomes in APS-RPL patients. This approach aims to provide a more comprehensive understanding of the immunological mechanisms underlying APS-RPL and offer more reliable evidence for clinical assessment and management.

## 2 Materials and methods

### 2.1 Study participants

This study was designed as a single-center retrospective analysis. The study group consisted of 153 women diagnosed with APS-RPL at Sichuan Jinxin Xinan Women and Children’s Hospital between 2020 and 2022.

#### 2.1.1 Inclusion criteria

The inclusion criteria were as follows:

(1) patients who met the definition of RPL as outlined by the ESHRE, (2) patients who met the clinical criteria for APS, (3) patients who underwent testing for aPS/PT antibodies and standard aPL antibodies, and (4) patients who underwent assisted reproductive treatment (*in vitro* fertilization, IVF).

#### 2.1.2 Exclusion criteria

(1) Patients with gynecological disorders, including endometritis, uterine fibroids, or hydrosalpinx; (2) patients with anatomical abnormalities of the reproductive tract, such as endometrial polyps, intrauterine adhesions, or congenital uterine malformations; (3) patients with genetic abnormalities, including those with abnormal embryonic karyotypes; (4) patients with confirmed systemic autoimmune diseases—such as systemic lupus erythematosus, Sjögren’s syndrome, systemic sclerosis, idiopathic inflammatory myopathies, or systemic vasculitis—as well as individuals clinically suspected of having systemic autoimmune disease (e.g., persistently high-titer antinuclear antibody (ANA) positivity accompanied by relevant clinical manifestations); and patients with isolated low-titer ANA positivity, without a definitive diagnosis or clinical features of systemic autoimmune disease, were not excluded; (5) patients with other systemic conditions known to contribute to RPL, including endocrine disorders, obesity, hypertension, cardiovascular disease, or renal disease; (6) patients with recurrent implantation failure (RIF); and (7) patients with a history of major adverse obstetric outcomes were excluded, including: (i) intrauterine fetal demise at ≥10 weeks of gestation in a prior pregnancy, (ii) severe preeclampsia occurring before 34 weeks of gestation, and (iii) previous pregnancies complicated by placental insufficiency resulting in adverse outcomes.

The control group included women who underwent *in vitro* fertilization (IVF) treatment at the same reproductive center during the same period and had a documented history of previous full-term normal delivery. All controls had no history of RPL, no venous or arterial thrombotic events, and no pregnancy complications meeting the diagnostic criteria for APS, such as fetal demise ≥10 weeks, severe preeclampsia, or placental insufficiency. In addition, none of the control participants had any known autoimmune diseases. All control subjects were tested for standard aPL antibodies and aPS/PT antibodies. None met the laboratory classification criteria for APS, defined as the absence of persistently high-titer aCL antibodies, aβ2GPI antibodies, LA, or triple antibody positivity. Baseline antibody levels in the control group were defined using the measurements obtained prior to their IVF treatment. For the study group, baseline values were based on antibody tests performed before the initiation of IVF and any APS-related therapy. All participants in both the study and control groups underwent IVF treatment at the same reproductive center, following a fully standardized clinical protocol.

*Ovarian stimulation protocols*: All patients received standardized controlled ovarian stimulation using either a gonadotropin-releasing hormone (GnRH) antagonist protocol or a long GnRH agonist protocol. The initial gonadotropin dose and subsequent adjustments were determined according to uniform criteria, taking into account patient age, ovarian reserve, and follicular response. When the leading follicles reached an appropriate size, oocyte maturation was triggered according to a fixed schedule, followed by transvaginal oocyte retrieval.

*Embryo culture and transfer strategy*: All embryos were cultured under identical laboratory conditions using a unified grading system. All patients underwent single blastocyst transfer. Endometrial preparation and luteal phase support were standardized across both groups, and embryo selection criteria were identical, ensuring full comparability of procedural factors.

#### 2.1.3 Patient group definitions

A total of 100 patients were ultimately included in the study based on the inclusion and exclusion criteria. Relevant clinical data and laboratory test results were collected, and patients were categorized into three groups according to pregnancy outcomes.

##### 2.1.3.1 Successful pregnancy group (*n* = 55)

The successful pregnancy group was defined as women who achieved a pregnancy ≥34 weeks and delivered a live-born infant without evidence of fetal growth restriction. In addition, no patients in this group experienced APS-related pregnancy complications, such as placental insufficiency, before 34 weeks of gestation.

##### 2.1.3.2 Pregnancy loss group (*n* = 35)

This group included patients who experienced recurrent pregnancy loss despite treatment. Pregnancy loss was defined as fetal demise before 20 weeks of gestation.

##### 2.1.3.3 Lost to follow-up group (*n* = 10)

This group included patients for whom complete follow-up data were not available after treatment.

*Treatment protocol in this study*: All patients in the study group (including both the successful pregnancy group and the pregnancy loss group) received a standardized anticoagulation regimen. Low-dose aspirin (75–100 mg/day) was administered to 100% of patients, initiated prior to ovarian stimulation or during the oocyte retrieval cycle, and continued throughout the pregnancy. Similarly, all patients received low-molecular-weight heparin (LMWH, 4,000–5,000 IU/day, subcutaneously), also with a 100% usage rate, starting at the initiation of luteal support and continuing for the duration of pregnancy. Immunomodulatory therapy was evaluated and adjusted by the same reproductive immunology team according to antibody profiles and clinical manifestations. Hydroxychloroquine (200 mg/day) was used in 31% of patients in the successful pregnancy group and 37% in the pregnancy loss group; treatment was initiated at least 1 month before ovarian stimulation and continued until approximately 12 weeks of gestation. Low-dose prednisone (5–10 mg/day) was used in 15% of the successful pregnancy group and 11% of the pregnancy loss group, initiated during the embryo transfer cycle or after a positive pregnancy test, and continued until 8–12 weeks of gestation. The specific clinical and treatment characteristics of the study population are outlined in Table 1.

**Table 1:** Baseline clinical characteristics and treatment regimens of the study population.

Characteristic	Study population (*n* = 100)	Successful pregnancy (*n* = 55)	Pregnancy loss (*n* = 35)	*p*-value
Recurrent pregnancy loss ≥3 episodes before 10 weeks	100	55	35	–
History of fetal demise (≥10 weeks)	0	0	0	–
Previous placental insufficiency	0	0	0	–
Prior severe preeclampsia (<34 weeks)	0	0	0	–
Use of low-dose aspirin	100 (100%)	55 (100%)	35 (100%)	–
Use of low-molecular-weight heparin	100 (100%)	55 (100%)	35 (100%)	–
Use of hydroxychloroquine	32 (32%)	17 (31%)	13 (37%)	0.702
Use of prednisone	13 (13%)	8 (15%)	4 (11%)	0.760

The *p*-values presented refer to comparisons between the successful pregnancy group and the pregnancy loss group.

### 2.2 Experimental methods

#### 2.2.1 Sample collection and processing

1. For the detection of aPS/PT IgM/IgG, aCL IgM/IgG/IgA, and aβ2GPI IgM/IgG/IgA, serum was separated using yellow-topped tubes. The serum was stored at 2–8 °C and should be tested within 72 h.
2. For lupus anticoagulant (LA) detection, serum was separated using blue-topped tubes. The serum was then transferred to bullet tubes and stored at below −20 °C. After thawing and returning to room temperature, testing was conducted within 96 h.
3. Timing of blood collection: Blood samples were collected during the luteal phase before treatment, again during the luteal phase 1 to 3 months after treatment, and during early pregnancy (5–8 weeks) for pregnancy-related blood sampling.

#### 2.2.2 Methods, instruments, and reagents

The detection of aPS/PT IgM/IgG and aβ2GPI IgM/IgG/IgA was performed using an enzyme-linked immunosorbent assay (ELISA) with manual procedures. The final results were read by an ELISA reader. The relevant reagents were purchased from Wofen Medical Equipment Trading Co., Ltd., and Omon Medical Diagnostics Co., Ltd.

The detection of aCL IgM/IgG/IgA was carried out using chemiluminescence, performed by a fully automated chemiluminescence immunoanalyzer (BIO-FLASH). Reagents were purchased from Wofen Medical Equipment Trading Co., Ltd.

Lupus anticoagulant (LA) detection was performed using the Dilute Russell’s Viper Venom Time (DRVVT) and silica clotting time (SCT) methods on a CS5100 automated coagulometer. Reagents were purchased from Siemens Medical Diagnostics GmbH, Germany.

### 2.3 Statistical analysis

Statistical analyses were performed using R software (version 4.2.2) and RStudio. A 2 × 3 mixed-design repeated measures ANOVA was conducted, with time (pre-treatment, post-treatment, and early pregnancy) as the within-subject factor and pregnancy outcome (successful vs. pregnancy loss) as the between-subject factor, to evaluate the main effects of time, group, and their interaction. When a significant overall time effect or time×group interaction was observed, one-way repeated measures ANOVA was performed within each group, followed by Bonferroni-corrected paired *t*-tests for pairwise comparisons between time points. In addition, independent-samples *t*-tests were used to compare differences between the successful pregnancy and pregnancy loss groups at each individual time point.

Continuous variables are presented as mean ± standard deviation (mean ± SD). All statistical tests were two-sided, and a *p*-value of < 0.01 was considered statistically significant.

## 3 Results

### 3.1 Baseline comparisons between the study group and the control group

There were no significant statistical differences in baseline characteristics, such as age, weight, height, body mass index (BMI), and menstrual cycle, between the study group and the control group (Table 2). Furthermore, there were no significant differences between the normal pregnancy group and the pregnancy loss group in terms of age, weight, height, BMI, menstrual cycle, and number of miscarriages (Table 3).

**Table 2:** Comparison of baseline characteristics between the study group and the control group.

Group	Study group (*n* = 100)	Control group (*n* = 30)	*P*-value
Age (years)	32.56 ± 4.51	31.43 ± 4.13	0.206
Weight (kg)	55.96 ± 5.09	55.01 ± 5.04	0.368
Height (m)	1.54 ± 0.05	1.54 ± 0.05	0.750
BMI	23.61 ± 2.55	23.37 ± 3.00	0.688
Menstrual cycle (days)	28.46 ± 3.48	29.73 ± 4.61	0.170

*P < 0.01, indicating statistical significance.

**Table 3:** Comparison of baseline characteristics between the successful pregnancy group and the pregnancy loss group.

Group	Normal pregnancy group (*n* = 55)	Pregnancy loss group (*n* = 35)	*P*-value
Age (years)	32.62 ± 4.58	32.17 ± 4.62	0.655
Weight (kg)	55.89 ± 4.89	56.57 ± 5.46	0.554
Height (m)	1.54 ± 0.04	1.54 ± 0.05	0.836
BMI	23.53 ± 2.36	23.92 ± 2.84	0.505
Menstrual cycle (days)	28.40 ± 4.00	28.49 ± 2.80	0.905
Number of pregnancy losses	3.15 ± 0.62	3.00 ± 0.64	0.292

*P < 0.01, indicating statistical significance.

### 3.2 Baseline clinical characteristics and treatment regimens of the study population

Table 1 summarizes the baseline clinical characteristics and treatment regimens of the study cohort. All enrolled patients met the diagnostic criteria for recurrent pregnancy loss (≥3 consecutive miscarriages before 10 weeks of gestation). No patient had a history of fetal death at ≥10 weeks, prior placental insufficiency, or severe preeclampsia diagnosed before 34 weeks.

All participants received standardized anticoagulation therapy, including low-dose aspirin and low-molecular-weight heparin. There were no significant differences between the successful pregnancy and pregnancy loss groups in the use of hydroxychloroquine (31% vs. 37%, *p* = 0.702) or prednisone (15% vs. 11%, *p* = 0.760).

### 3.3 Comparison between the study and control groups

Serum aPS/PT IgM and aCL IgG in the study group were significantly higher than those in the control group (Table 4, *P* < 0.01). However, no significant differences were observed between the two groups for other antibodies, including aβ2GPI IgM/IgG/IgA, aCLIgM/IgA, aPS/PT IgG, and LA.

**Table 4:** Comparison of standard aPL antibodies and aPS/PT antibodies between the study group and the control group.

Group	aCL IgG (CU)	aCL IgM (CU)	aCL IgA (CU)	aβ2GPI IgG (RU/mL)	aβ2GPI IgM (RU/mL)	aβ2GPI IgA (RU/mL)	LA1 (sec)	LA2 (sec)	LA1 (SCT) (sec)	LA2 (SCT) (sec)	aPS/PT IgG (U/mL)	aPS/PT IgM (U/mL)
Study group (*n* = 100)	16.39 ± 3.63	10.7 ± 3.57	10.35 ± 4.47	12.69 ± 4.52	14.11 ± 3.82	7.46 ± 3.55	35.75 ± 3.63	33.37 ± 3.46	58.42 ± 4.96	36.54 ± 3.65	12.24 ± 3.76	48.73 ± 5.07
Control group (*n* = 30)	12.53 ± 5.24	11.26 ± 3.8	9.89 ± 3.84	11.95 ± 2.85	12.67 ± 4.73	8.14 ± 2.78	37.1 ± 4.11	34.16 ± 2.29	58.79 ± 4.77	35.64 ± 4.21	12.78 ± 3.32	10.89 ± 5.83
*P*-value	5.45 × 10^−4^*	0.477	0.589	0.284	0.135	0.273	0.114	0.151	0.715	0.299	0.456	<2.2 × 10^−16^*

*P < 0.01, indicating statistical significance.

### 3.4 Results of the 2 × 3 mixed-design repeated measures ANOVA

The 2 × 3 mixed-design repeated measures ANOVA showed significant time effects for aCL IgG, aβ2GPI IgM, and aPS/PT IgM across the three time points (pre-treatment, post-treatment, and early pregnancy) (Table 5, *P* < 0.01). Significant group effects were also observed for LA1, LA1-SCT, aPS/PT IgG, and aPS/PT IgM, indicating marked differences between the successful pregnancy and pregnancy loss groups (Table 5, *P* < 0.01). In addition, both aPS/PT IgG and aPS/PT IgM demonstrated significant time × group interactions (Table 5, *P* < 0.01), reflecting distinct dynamic trajectories between the two outcome groups.

**Table 5:** Mixed-design repeated measures ANOVA for antibody levels.

Antibody	Group effect F	*P* (group)	Time effect F	*P* (time)	Group × time F	*P* (interaction)
aCL IgG	4.71	0.033	44.23	<0.001*	0.34	0.714
aCL IgM	0.05	0.828	0.12	0.884	1.23	0.295
aCL IgA	2.17	0.144	0.41	0.663	0.88	0.417
aβ2GPI IgG	0.80	0.374	1.53	0.220	0.98	0.377
aβ2GPI IgM	5.03	0.028	11.35	<0.001*	1.69	0.187
aβ2GPI IgA	1.28	0.262	0.35	0.709	1.02	0.364
LA1	10.65	0.002*	3.67	0.027	0.24	0.789
LA2	6.36	0.013	0.79	0.456	0.49	0.616
LA1 (SCT)	8.86	0.004*	0.58	0.562	0.95	0.391
LA2 (SCT)	0.39	0.533	2.32	0.101	0.79	0.454
aPS/PT IgG	11.48	0.001*	1.42	0.244	9.28	<0.001*
aPS/PT IgM	1899.52	<0.001*	403.46	<0.001*	483.95	<0.001*

Values are F statistics from mixed-design repeated measures ANOVA. df for group = (1, 88); df for time and group × time = (2, 176). *P < 0.01, indicating statistical significance.

### 3.5 Within-group repeated measures ANOVA with Bonferroni-corrected pairwise comparisons

**Figure 1:**
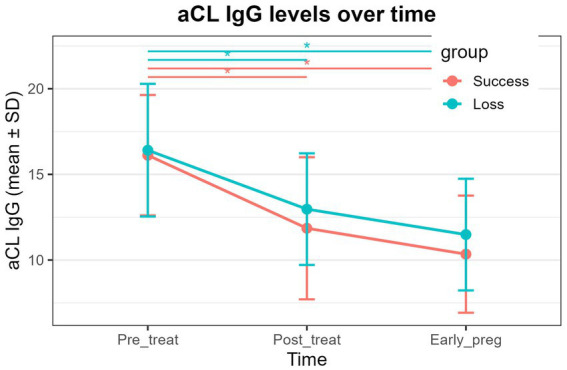
Dynamic changes in aCL IgG levels in the successful pregnancy group and the pregnancy loss group at three time points (pre-treatment, post-treatment, and early pregnancy). The lines represent group mean values over time, with error bars indicating standard deviations. Asterisks above the plot denote statistically significant pairwise comparisons between time points (*P* < 0.01).

**Figure 2:**
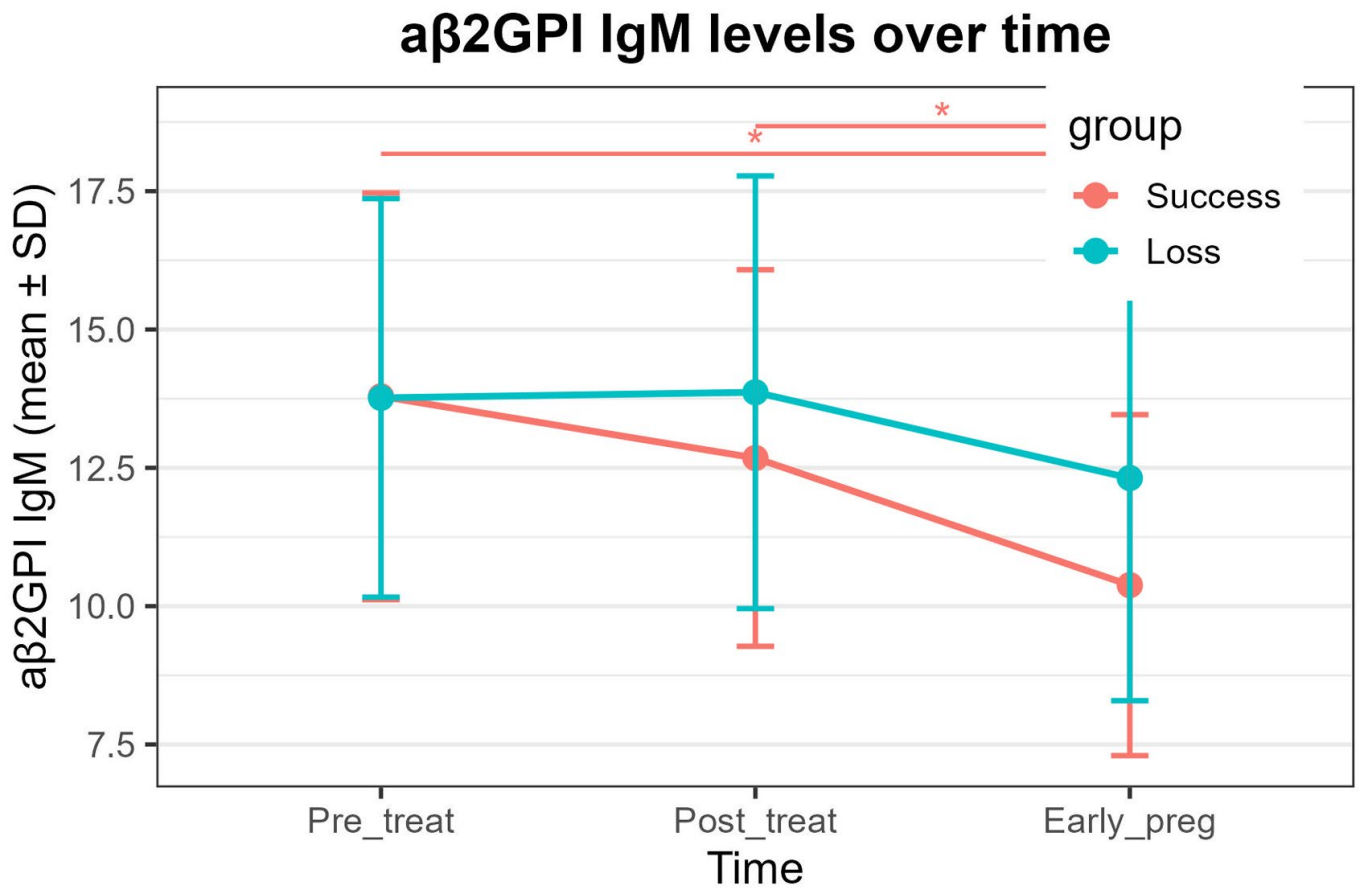
Dynamic changes in aβ2GPI IgM levels in the successful pregnancy group and the pregnancy loss group at three time points (pre-treatment, post-treatment, and early pregnancy). The lines represent group mean values over time, with error bars indicating standard deviations. Asterisks above the plot denote statistically significant pairwise comparisons between time points (*P* < 0.01).

**Figure 3:**
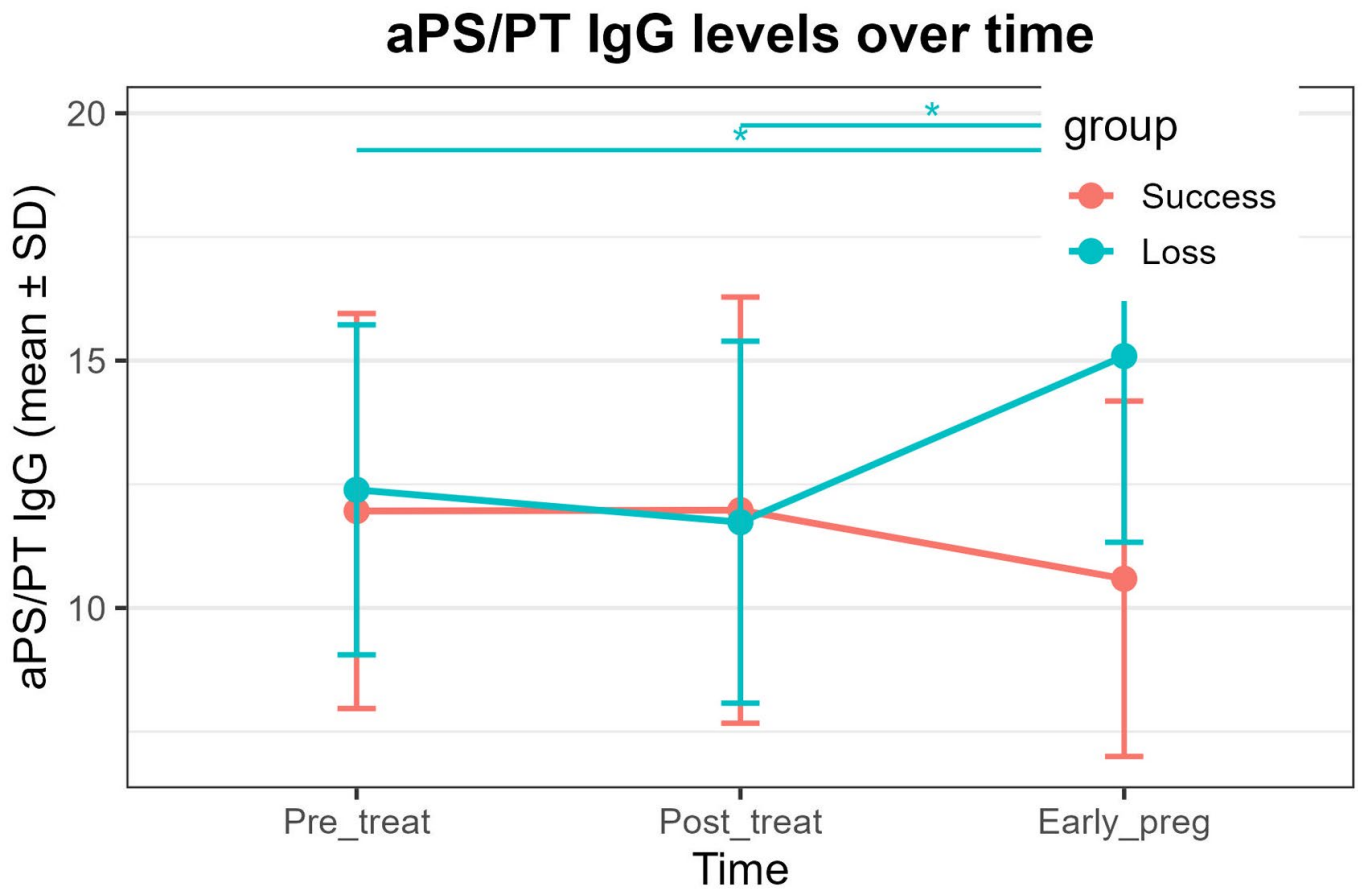
Dynamic changes in aPS/PT IgG levels in the successful pregnancy group and the pregnancy loss group at three time points (pre-treatment, post-treatment, and early pregnancy). The lines represent group mean values over time, with error bars indicating standard deviations. Asterisks above the plot denote statistically significant pairwise comparisons between time points (*P* < 0.01).

**Figure 4:**
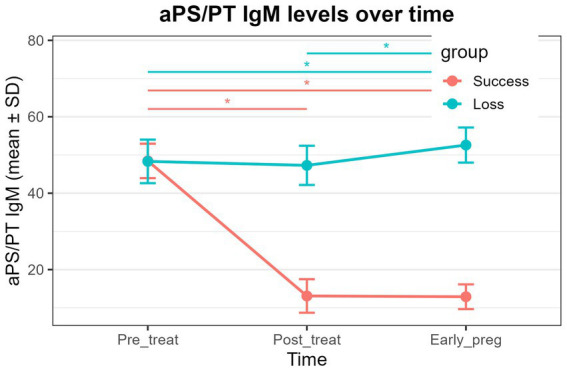
Dynamic changes in aPS/PT IgM levels in the successful pregnancy group and the pregnancy loss group at three time points (pre-treatment, post-treatment, and early pregnancy). The lines represent group mean values over time, with error bars indicating standard deviations. Asterisks above the plot denote statistically significant pairwise comparisons between time points (*P* < 0.01).

1. aCL IgG: Both the successful pregnancy group and the pregnancy loss group exhibited significant time effects (Table 6, *P* < 0.01). In both groups, aCL IgG levels at post-treatment and early pregnancy were significantly lower than pre-treatment levels (Figure 1 and Table 6, *P* < 0.01), whereas no significant difference was observed between the post-treatment and early-pregnancy time points.
2. aβ2GPI IgM: A significant time effect was detected in the successful pregnancy group (Table 6, *P* < 0.01), with early-pregnancy levels significantly lower than both pre-treatment and post-treatment levels (Figure 2 and Table 6, *P* < 0.01). No significant temporal changes were observed in the pregnancy loss group.
3. aPS/PT IgG: A significant time effect was observed only in the pregnancy loss group (Table 6, *P* < 0.01). Early-pregnancy aPS/PT IgG levels were significantly higher than levels at both pre-treatment and post-treatment (Figure 3 and Table 6, *P* < 0.01). No significant temporal differences occurred in the successful pregnancy group. In addition, aPS/PT IgG levels showed minimal changes after treatment in both groups; however, the successful pregnancy group exhibited a slight decline in early pregnancy, whereas the pregnancy loss group demonstrated a marked increase (Table 6 and Figure 3). Consequently, the two groups displayed distinctly different patterns of change during early pregnancy (Table 5 and Figure 3, P interaction < 0.001).
4. aPS/PT IgM: Both groups showed significant time effects (Table 6, *P* < 0.01), but the directions of change differed. In the successful pregnancy group, aPS/PT IgM levels at post-treatment and early pregnancy were significantly lower than pre-treatment levels (Figure 4 and Table 6, *P* < 0.01). In contrast, in the pregnancy loss group, early-pregnancy levels were significantly higher than both pre-treatment and post-treatment levels (Figure 4 and Table 6, *P* < 0.01). Moreover, aPS/PT IgM levels in the successful pregnancy group declined rapidly after treatment and remained consistently low thereafter (Table 6 and Figure 4). In contrast, the pregnancy loss group showed minimal change following treatment but exhibited a marked increase during early pregnancy (Table 6 and Figure 4), resulting in a dynamic trajectory that differed significantly from that of the successful pregnancy group (Table 5 and Figure 4, P interaction < 0.001).

**Table 6:** Within-group repeated measures ANOVA and Bonferroni-adjusted pairwise comparisons for antibody levels.

Antibodies	Group	Pre-treatment	Post-treatment	Post-pregnancy	Time effect (RM-ANOVA *p*)	Pre-treatment vs. post-treatment	Pre-treatment vs. post-pregnancy	Post-treatment vs. post-pregnancy
aCL IgG	Success	16.12 ± 3.52	11.85 ± 4.15	10.34 ± 3.42	**6.33 × 10**^−12^*	1.1 × 10^−6^*	8.5 × 10^−10^*	0.13
	Loss	16.41 ± 3.87	12.97 ± 3.26	11.49 ± 3.26	**2.89 × 10**^−6^*	0.001*	3 × 10^−5^*	0.331
aβ2GPI IgM	Success	13.79 ± 3.67	12.68 ± 3.40	10.38 ± 3.08	**1.57 × 10**^−6^*	0.209	6.2 × 10^−6^*	0.0025*
	Loss	13.76 ± 3.60	13.87 ± 3.91	12.32 ± 4.02	0.174	1.00	0.45	0.25
aPS/PT IgG	Success	11.96 ± 3.99	11.98 ± 4.31	10.59 ± 3.59	0.144	1.00	0.28	0.28
	Loss	12.39 ± 3.34	11.73 ± 3.66	15.09 ± 3.76	**1.78 × 10**^−4^*	1.00	0.0033*	0.0013*
aPS/PT IgM	Success	48.44 ± 4.51	13.10 ± 4.39	12.89 ± 3.24	**9.24 × 10**^−79^*	<2 × 10^−16^*	<2 × 10^−16^*	1.00
	Loss	48.32 ± 5.70	47.28 ± 5.13	52.59 ± 4.60	**1.68 × 10**^−4^*	1.00	0.0063*	4.9 × 10^−5^*

*p* values marked with an asterisk (*) in the Time Effect (RM-ANOVA p) column indicate that changes in antibody levels across the three within-group time points (pre-treatment, post-treatment, and post-pregnancy) reached statistical significance after Bonferroni adjustment (*p* < 0.01).

### 3.6 Comparison between the pregnancy loss group and successful pregnancy group

The pregnancy loss group exhibited significantly higher aPS/PT IgM levels both after treatment and during early pregnancy compared with the successful pregnancy group (Table 7, *p* < 0.01). In addition, aPS/PT IgG levels during early pregnancy were also markedly higher in the pregnancy loss group (Table 7, *p* < 0.01). Apart from these differences, no significant variations were observed between the two groups across the three time points for the remaining antibodies, including β2GPI IgM/IgG/IgA, aCL IgM/IgG/IgA, and LA.

**Table 7:** The pre-treatment and post-treatment and post-pregnancy comparison of standard APL antibodies and APS/PT antibodies between the pregnancy loss group and the successful pregnancy group.

Group	aCL IgG (CU)	aCL IgM (CU)	aCL IgA (CU)	aβ2GPI IgG (RU/mL)	aβ2GPI IgM (RU/mL)	aβ2GPI IgA (RU/mL)	LA1 (sec)	LA2 (sec)	LA1 (SCT) (sec)	LA2 (SCT) (sec)	aPS/PT IgG (U/mL)	aPS/PT IgM (U/mL)
Pregnancy group, pre-treatment (*n* = 55)	16.12 ± 3.52	10.43 ± 3.78	10.50 ± 4.73	12.56 ± 4.05	13.79 ± 3.67	7.36 ± 3.48	35.02 ± 4.03	33.47 ± 3.37	59.52 ± 4.97	36.31 ± 3.77	11.96 ± 3.99	48.44 ± 4.51
Loss group, pre-treatment (*n* = 35)	16.41 ± 3.87	11.48 ± 3.12	10.42 ± 4.49	12.76 ± 4.55	13.76 ± 3.60	7.29 ± 3.58	36.59 ± 2.57	32.90 ± 3.28	56.82 ± 4.56	36.58 ± 3.74	12.39 ± 3.34	48.32 ± 5.70
P1 value	0.717	0.160	0.941	0.836	0.971	0.927	0.026	0.423	0.010	0.738	0.584	0.913
Pregnancy group, post-treatment (*n* = 55)	11.85 ± 4.15	10.86 ± 3.94	9.15 ± 4.53	12.32 ± 3.37	12.68 ± 3.4	8.26 ± 2.8	34.13 ± 3.26	34.16 ± 3.39	59.44 ± 4.35	36.39 ± 3.6	11.98 ± 4.31	13.10 ± 4.39
Loss group, post-treatment (*n* = 35)	12.97 ± 3.26	10.54 ± 2.19	10.68 ± 2.48	10.94 ± 4.50	13.87 ± 3.91	6.96 ± 3.18	35.10 ± 2.99	32.66 ± 3.97	58.26 ± 3.04	35.27 ± 4.36	11.73 ± 3.66	47.28 ± 5.13
P2 value	0.159	0.627	0.042	0.124	0.145	0.052	0.153	0.069	0.132	0.209	0.775	<2.2 × 10^−16^*
Pregnancy group, post-pregnancy (*n* = 55)	10.34 ± 3.42	10.99 ± 4.02	9.87 ± 2.97	12.32 ± 3.72	10.38 ± 3.08	7.19 ± 3.81	33.76 ± 3.91	34.40 ± 2.96	58.63 ± 4.88	35.15 ± 3.82	10.59 ± 3.59	12.89 ± 3.24
Loss group, post-pregnancy (*n* = 35)	11.49 ± 3.26	10.54 ± 1.82	10.56 ± 3.33	12.18 ± 3.37	12.32 ± 4.02	7.20 ± 2.77	35.36 ± 2.58	33.17 ± 2.59	57.77 ± 4.83	35.19 ± 3.05	15.09 ± 3.76	52.59 ± 4.60
P3 value	0.116	0.470	0.326	0.858	0.018	0.986	0.022	0.040	0.412	0.960	3.48 × 10^−7^*	<2.2 × 10^−16^*

In this table, the P1 value represents the pre-treatment comparison between the successful pregnancy group and the pregnancy loss group; the P2 value represents the post-treatment comparison between the successful pregnancy group and the pregnancy loss group; the P3 value represents the post-pregnancy comparison between the successful pregnancy group and the pregnancy loss group. *P < 0.01, indicating statistical significance.

## 4 Discussion

This study systematically evaluated the dynamic changes of standard aPL antibodies and aPS/PT antibodies in APS-RPL patients before treatment, after treatment, and during early pregnancy, with the aim of elucidating the potential immunomodulatory mechanisms underlying pregnancy maintenance. By comparing the temporal antibody profiles between women with successful pregnancies and those who experienced pregnancy loss, we sought to investigate the association between immune dynamics and pregnancy outcomes from a longitudinal immunological perspective. Unlike traditional analyses focusing on antibody levels at a single time point, this study highlights the importance of antibody trajectories and group-specific differences over time. Such an approach provides valuable insights into the evolving immune responses associated with APS and their clinical implications while also offering new avenues for identifying high-risk individuals and optimizing pregnancy management strategies.

Previous studies have shown that aPS/PT antibodies have high diagnostic values in Chinese aPS patients and serve as important biomarkers and risk predictors for thrombosis and pregnancy loss (17). Elevated aPS/PT IgM levels in APS and RPL populations are strongly associated with adverse pregnancy outcomes (11, 12), and their sensitivity appears to exceed that of aCL and aβ2GPI antibodies (21). As one of the standard aPL antibodies, aCL—particularly aCL IgG—is well-recognized for its association with an increased risk of spontaneous miscarriage (22, 23). In line with these findings, our study demonstrated significantly higher levels of aPS/PT IgM and aCL IgG in the study group compared with the control group, suggesting that both antibodies may represent important immunologic contributors to heightened miscarriage risk. By contrast, no significant differences were observed in aβ2GPI antibodies or LA levels between the two groups. Although these markers are conventionally linked to thrombosis and pregnancy complications (24–26), the lack of association in our study may reflect factors such as limited sample size, substantial inter-individual variability in antibody levels, and differences in assay sensitivity across platforms (27, 28).

The mixed-design repeated measures ANOVA revealed heterogeneous patterns across different antibodies with respect to time effects, group effects, and their interactions. Among the standard aPL antibodies, aCL IgG and aβ2GPI IgM exhibited significant time effects, suggesting that immunotherapy and physiological changes during pregnancy may modulate their levels to varying degrees, whereas other standard antibodies showed no notable temporal fluctuations. Notably, both aPS/PT IgG and aPS/PT IgM exhibited significant group effects and time × group interactions, indicating that their temporal trajectories differed markedly between the successful pregnancy and pregnancy loss groups. These findings imply that, compared with traditional aPL antibodies, aPS/PT antibodies may possess greater sensitivity and clinical value in reflecting pregnancy-related immune alterations and predicting pregnancy outcomes (11). This observation provides a foundation for subsequent analyses of antibody dynamics and their clinical implications.

Further within-group repeated measures analyses revealed that the successful pregnancy group displayed a more consistent downward trend across multiple antibodies over time. Levels of aCL IgG, aβ2GPI IgM, and aPS/PT IgM were all significantly lower in early pregnancy compared with pre-treatment levels. This decline aligns with previous hypotheses proposing that “post-treatment immune burden reduction and early pregnancy-associated immune tolerance” contribute to favorable pregnancy outcomes (29, 30). In contrast, the pregnancy loss group exhibited distinctly different temporal patterns: although aCL IgG declined after treatment, aPS/PT IgG and aPS/PT IgM were not adequately suppressed and instead rose substantially during early pregnancy. Particularly for aPS/PT IgM, levels decreased in the successful group but increased in the loss group, demonstrating opposite directions of change. These divergent patterns suggest that whereas reductions in standard aPL antibodies may reflect partial treatment effects, the dynamic profiles of aPS/PT antibodies more accurately capture the success or failure of early pregnancy-related immune regulation.

Between-group comparisons across the three time points further highlighted the discriminative value of aPS/PT antibodies. Although baseline antibody levels were largely comparable between the groups, the pregnancy loss group showed significantly higher aPS/PT IgM levels after treatment and during early pregnancy, accompanied by an early-pregnancy elevation in aPS/PT IgG. Moreover, while the successful pregnancy group exhibited an overall decline or stabilization of immune markers in early pregnancy, the loss group displayed persistent or even exacerbated immune burden. These findings indicate that baseline antibody levels alone are insufficient to predict pregnancy outcomes; instead, the dynamic trajectories—particularly those of aPS/PT IgM and aPS/PT IgG—may serve as more informative immunological indicators. This conclusion is consistent with earlier reports identifying aPS/PT antibodies as risk-associated markers and further supports their potential clinical utility as predictors of adverse pregnancy outcomes (18–20).

This study has several limitations. First, as a retrospective analysis, inherent biases in data collection and documentation cannot be entirely avoided. Second, the sample size is relatively limited; although the results demonstrated highly consistent trends, their generalizability requires confirmation in larger, multicenter cohorts. In addition, variations in sensitivity and specificity across different detection platforms may introduce methodological variability. Finally, because low-titer ANA-positive patients were not stratified separately, the potential influence of ANA status on certain immune parameters cannot be completely excluded. Despite these limitations, the present study offers several notable strengths. To the best of our knowledge, this is the first investigation to use a longitudinal self-comparison design, evaluating dynamic changes in antibody levels at three key time points—before treatment, after treatment, and during early pregnancy—within both the successful pregnancy and pregnancy loss groups. In contrast to previous studies that predominantly relied on cross-sectional analyses, this three-time-point within-subject approach effectively minimizes inter-individual variability and strengthens the reliability of interpreting true immunological trajectories. Moreover, by applying a mixed-design repeated measures ANOVA, we systematically assessed time effects, group effects, and their interactions. The results clearly revealed significant time × group interactions for both aPS/PT IgG and aPS/PT IgM, with the two groups exhibiting opposite temporal trends. This pronounced immunodynamic divergence has not been adequately characterized in prior literature, underscoring the unique contribution of our study design. Complementing this, three-time-point between-group comparisons further revealed that the pregnancy loss group consistently maintained elevated levels of aPS/PT IgM and IgG after treatment and during early pregnancy, whereas the successful pregnancy group showed marked reductions or stabilization. These findings provide compelling evidence linking differential immune regulation to pregnancy outcomes.

Looking ahead, we plan to expand the sample size and conduct multicenter studies to enhance the robustness and generalizability of our conclusions. Additionally, integrating multi-platform testing and incorporating other immunologic markers—such as stratified ANA analyses—may offer a more comprehensive understanding of the maternal immune environment in pregnancy maintenance. Such efforts are expected to further support the development of more precise immunological intervention strategies for patients with recurrent pregnancy loss.

## Data Availability

Publicly available datasets were analyzed in this study. This data can be found here: all data will be made available upon reasonable request to and with the approved consent of the corresponding author and will be shared in accordance with the standards of ethical policies regulating data sharing in human subjects.

## References

[1] Rai R Regan L. Recurrent miscarriage. Lancet. (2006) 368:601–11. doi: 10.1016/S0140-6736(06)69204-0, 16905025

[2] Practice Committee of the American Society for Reproductive Medicine. Evaluation and treatment of recurrent pregnancy loss: a committee opinion. Fertil Steril. (2012) 98:1103–11. doi: 10.1016/j.fertnstert.2012.06.048, 22835448

[3] Ford HB Schust DJ. Recurrent pregnancy loss: etiology, diagnosis, and therapy. Rev Obstet Gynecol. (2009) 2:76–83. 19609401 PMC2709325

[4] Vomstein K Feil K Strobel L Aulitzky A Hofer-Tollinger S Kuon RJ . Immunological risk factors in recurrent pregnancy loss: guidelines versus current state of the art. J Clin Med. (2021) 10:1–21. doi: 10.3390/jcm10040869, 33672505 PMC7923780

[5] Hennessy M Dennehy R Meaney S Devane D O'Donoghue K. A protocol for a systematic review of clinical practice guidelines for recurrent miscarriage. HRB Open Res. (2020) 3:12. doi: 10.12688/hrbopenres.13024.3, 33005862 PMC7477641

[6] Pham M Orsolini G Crowson C Snyder M Pruthi R Moder K. Anti-phosphatidylserine prothrombin antibodies as a predictor of the lupus anticoagulant in an all-comer population. J Thromb Haemost. (2022) 20:2070–4. doi: 10.1111/jth.15792, 35722911

[7] Garcia D Erkan D. Diagnosis and management of the antiphospholipid syndrome. N Engl J Med. (2018) 378:2010–21. doi: 10.1056/NEJMra1705454, 29791828

[8] Cervera R Serrano R Pons-Estel GJ Ceberio-Hualde L Shoenfeld Y de Ramón E . Morbidity and mortality in the antiphospholipid syndrome during a 10-year period: a multicentre prospective study of 1000 patients. Ann Rheum Dis. (2015) 74:1011–8. doi: 10.1136/annrheumdis-2013-204838, 24464962

[9] Shetty S Ghosh K. Anti-phospholipid antibodies and other immunological causes of recurrent foetal loss--a review of literature of various therapeutic protocols. Am J Reprod Immunol. (2009) 62:9–24. doi: 10.1111/j.1600-0897.2009.00714.x 19527228

[10] Miyakis S Lockshin MD Atsumi T Branch DW Brey RL Cervera R . International consensus statement on an update of the classification criteria for definite antiphospholipid syndrome (APS). J Thromb Haemost. (2006) 4:295–306. doi: 10.1111/j.1538-7836.2006.01753.x, 16420554

[11] Xiang J Pan Y Bao R Cai Z. Correlation of anti-phosphatidylserine/prothrombin and anti-phosphatidylserine antibodies with pregnancy outcomes. Am J Reprod Immunol. (2024) 92:e13890. doi: 10.1111/aji.13890, 38958240

[12] Pleguezuelo DE Cabrera-Marante O Abad M Rodriguez-Frias EA Naranjo L Vazquez A . Anti-phosphatidylserine/prothrombin antibodies in healthy women with unexplained recurrent pregnancy loss. J Clin Med. (2021) 10:2094. doi: 10.3390/jcm10102094, 34068095 PMC8152729

[13] Nojima J Iwatani Y Suehisa E Kuratsune H Kanakura Y. The presence of anti-phosphatidylserine/prothrombin antibodies as risk factor for both arterial and venous thrombosis in patients with systemic lupus erythematosus. Haematologica. (2006) 91:699–702. 16627257

[14] Vlagea A Gil A Cuesta MV Arribas F Diez J Lavilla P . Antiphosphatidylserine/prothrombin antibodies (aPS/PT) as potential markers of antiphospholipid syndrome. Clin Appl Thromb Hemost. (2013) 19:289–96. doi: 10.1177/1076029612437578, 22387581

[15] Sciascia S Sanna G Murru V Roccatello D Khamashta MA Bertolaccini ML. Anti-prothrombin (aPT) and anti-phosphatidylserine/prothrombin (aPS/PT) antibodies and the risk of thrombosis in the antiphospholipid syndrome. A systematic review. Thromb Haemost. (2014) 112:354–64. doi: 10.1160/TH13-06-0509 24172938

[16] Radin M Foddai SG Cecchi I Rubini E Schreiber K Roccatello D . Antiphosphatidylserine/prothrombin antibodies: an update on their association with clinical manifestations of antiphospholipid syndrome. Thromb Haemost. (2020) 120:592–8. doi: 10.1055/s-0040-1705115, 32185783

[17] Shi H Zheng H Yin YF Hu QY Teng JL Sun Y . Antiphosphatidylserine/prothrombin antibodies (aPS/PT) as potential diagnostic markers and risk predictors of venous thrombosis and obstetric complications in antiphospholipid syndrome. Clin Chem Lab Med. (2018) 56:614–24. doi: 10.1515/cclm-2017-0502, 29166262

[18] Yao ZQ Li CH Li XY Guo W Zhai JY Liu R . Correlation of anti-phosphatidylserine/prothrombin antibodies with unexplained recurrent miscarriages. Beijing Da Xue Xue Bao. (2023) 55:1058–61. doi: 10.19723/j.issn.1671-167X.2023.06.016, 38101789 PMC10724005

[19] Akimoto T Akama T Saitoh M Kono I Sumida T. Antiprothrombin autoantibodies in severe preeclampsia and abortion. Am J Med. (2001) 110:188–91. doi: 10.1016/s0002-9343(00)00694-x, 11182104

[20] Žigon P Perdan Pirkmajer K Tomšič M Kveder T Božič B Sodin Šemrl S . Anti-phosphatidylserine/prothrombin antibodies are associated with adverse pregnancy outcomes. J Immunol Res. (2015) 2015:975704. doi: 10.1155/2015/975704, 26078985 PMC4452858

[21] Heikal NM Jaskowski TD Malmberg E Lakos G Branch DW Tebo AE. Laboratory evaluation of anti-phospholipid syndrome: a preliminary prospective study of phosphatidylserine/prothrombin antibodies in an at-risk patient cohort. Clin Exp Immunol. (2015) 180:218–26. doi: 10.1111/cei.12573, 25522978 PMC4408156

[22] Velayuthaprabhu S Archunan G. Evaluation of anticardiolipin antibodies and antiphosphatidylserine antibodies in women with recurrent abortion. Indian J Med Sci. (2005) 59:347–52. doi: 10.4103/0019-5359.16651, 16129928

[23] Sater MS Finan RR Abu-Hijleh FM Abu-Hijleh TM Almawi WY. Anti-phosphatidylserine, anti-cardiolipin, anti-β2 glycoprotein I and anti-prothrombin antibodies in recurrent miscarriage at 8-12 gestational weeks. Eur J Obstet Gynecol Reprod Biol. (2012) 163:170–4. doi: 10.1016/j.ejogrb.2012.04.001, 22555404

[24] Sailer T Zoghlami C Kurz C Rumpold H Quehenberger P Panzer S . Anti-beta2-glycoprotein I antibodies are associated with pregnancy loss in women with the lupus anticoagulant. Thromb Haemost. (2006) 95:796–801. 16676070

[25] Liu XL Xiao J Zhu F. Anti-β2 glycoprotein I antibodies and pregnancy outcome in antiphospholipid syndrome. Acta Obstet Gynecol Scand. (2013) 92:234–7. doi: 10.1111/aogs.12038, 23157457

[26] Opatrny L David M Kahn SR Shrier I Rey E. Association between antiphospholipid antibodies and recurrent fetal loss in women without autoimmune disease: a meta analysis. J Rheumatol. (2006) 33:2214–21. 17014001

[27] Chayoua W Kelchtermans H Moore GW Moore G Gris J-C Musial J . Detection of anti-cardiolipin and anti-β2glycoprotein I antibodies differs between platforms without influence on association with clinical symptoms. Thromb Haemost. (2019) 119:797–806. doi: 10.1055/s-0039-1679901, 30822809

[28] Meneghel L Ruffatti A Gavasso S Tonello M Mattia E Spiezia L . The clinical performance of a chemiluminescent immunoassay in detecting anti-cardiolipin and anti-β2 glycoprotein I antibodies. A comparison with a homemade ELISA method. Clin Chem Lab Med. (2015) 53:1083–9. doi: 10.1515/cclm-2014-0925, 25720075

[29] Al-Balushi MS Hasson SS Said EA Al-Busaidi JZ Al-Daihani MS Othman MS . Fluctuation in the levels of immunoglobulin M and immunoglobulin G antibodies for cardiolipin and β2-glycoprotein among healthy pregnant women. Sultan Qaboos Univ Med J. (2014) 14:e478–e485. doi: 10.18295/2075-0528.1622, 25364550 PMC4205059

[30] Song Y Wang HY Qiao J Liu P Chi HB. Antiphospholipid antibody titers and clinical outcomes in patients with recurrent miscarriage and antiphospholipid antibody syndrome: a prospective study. Chin Med J. (2017) 130:267–72. doi: 10.4103/0366-6999.198934, 28139508 PMC5308007

